# Individualized targeted glucose control to avoid hypoglycemia

**DOI:** 10.1186/cc12394

**Published:** 2013-03-19

**Authors:** SP Gawel, G Clermont, T Ho, BM Newman, B Yegneswaran, RS Parker

**Affiliations:** 1 University of Pittsburgh, PA, USA; 2 University of Pittsburgh Medical Center, Pittsburgh, PA, USA; 3 Iowa State University, Ames, IA, USA

## Introduction

Hyperglycemia and hypoglycemia have been linked to worse outcomes in critically ill patients. While there is controversy as to the optimal tightness of glucose control in critically ill patients, there is agreement that an upper limit to safe glucose levels exists and that avoiding hypoglycemic episodes should be prioritized. Our algorithm can assist clinicians in maintaining blood glucose ([Gbl]) within a desired target range while avoiding hypoglycemia.

## Methods

Our model predictive control (MPC) algorithm uses insulin and glucose as control inputs and a linearized model of glucose-insulin-fatty acid interactions. To allow the controller model to learn from data, a moving horizon estimation (MHE) technique tailored the tissue sensitivity to insulin to individual responses. Patient data ([Gbl] measurements, insulin and nutritional infusion rates) were from the HIDENIC database at the University of Pittsburgh Medical Center. [Gbl] measurements, typically hourly, were interpolated to impute a measurement every 5 minutes. The model captured patient [Gbl] via nonlinear least squares by adjusting insulin sensitivity (SI) and endogenous glucose production (EGP0). The resulting virtual patient (VP) is used to evaluate the performance of the MPC-MHE algorithm.

## Results

**Figure 1:**
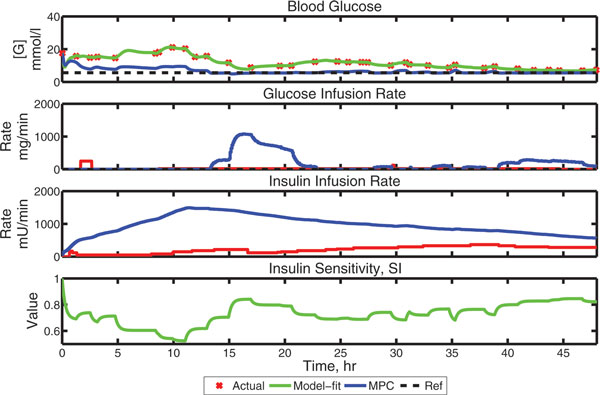


MPC controller performance on one VP is shown in Figure 1. Across a population of 10 VPs, the average [Gbl] under MPC is 6.31 mmol/l, the average minimum is 4.62 mmol/l, the population individual minimum is 3.49 mmol/l and the average absolute average residual error is 0.83 mmol/l from a 5.6 mmol/l target. With standard intervention, the 10 VPs have an average [Gbl] of 9.32 mmol/l, an average minimum [Gbl] of 3.77 mmol/l, and a population minimum [Gbl] of 2.78 mmol/l. Algorithm performance deteriorates significantly if the imputed sampling time exceeds 30 minutes, underlining the importance of dynamic variations in insulin sensitivity in this population.

## Conclusion

The MPC-MHE algorithm achieves targeted glucose control in response to changing patient dynamics and multiple measured disturbances for a pilot population of 10 VPs. Furthermore, the MHE scheme updates patient parameters in real time in response to changing patient dynamics.

